# A Live-Attenuated Prime, Inactivated Boost Vaccination Strategy with Chimeric Hemagglutinin-Based Universal Influenza Virus Vaccines Provides Protection in Ferrets: A Confirmatory Study

**DOI:** 10.3390/vaccines6030047

**Published:** 2018-07-25

**Authors:** Raffael Nachbagauer, Florian Krammer, Randy A. Albrecht

**Affiliations:** 1 Department of Microbiology, Icahn School of Medicine at Mount Sinai, New York, NY 10029, USA; raffael.nachbagauer@mssm.edu; 2 Global Health and Emerging Pathogens Institute, Icahn School of Medicine at Mount Sinai, New York, NY 10029, USA

**Keywords:** universal influenza virus vaccine, ferret model, chimeric hemagglutinin, humoral immunity

## Abstract

Influenza viruses cause severe diseases and mortality in humans on an annual basis. The current influenza virus vaccines can confer protection when they are well-matched with the circulating strains. However, due to constant changes of the virus surface glycoproteins, the vaccine efficacy can drop substantially in some seasons. In addition, the current seasonal influenza virus vaccines do not protect from avian influenza viruses of human pandemic potential. Novel influenza virus vaccines that aim to elicit antibodies against conserved epitopes like the hemagglutinin stalk could not only reduce the burden of drifted seasonal viruses but potentially also protect humans from infection with zoonotic and emerging pandemic influenza viruses. In this paper, we generated influenza virus vaccine constructs that express chimeric hemagglutinins consisting of exotic, avian head domains and a consistent stalk domain of a seasonal virus. Using such viruses in a sequential immunization regimen can redirect the immune response towards conserved epitopes. In this study, male ferrets received a live-attenuated vaccine virus based on the A/Ann Arbor/6/60 strain expressing a chimeric H8/1 (cH8/1) hemagglutinin, which was followed by a heterologous booster vaccination with a cH5/1N1 formalin inactivated non-adjuvanted whole virus. This group was compared to a second group that received a cH8/1N1 inactivated vaccine followed by a cH5/1N1 inactivated vaccine. Both groups showed a reduction in viral titers in the upper respiratory tract after the A(H1N1)pdm09 virus challenge. Animals that received the live-attenuated vaccine had low or undetectable titers in the lower respiratory tract. The results support the further development of chimeric hemagglutinin-based vaccination strategies. The outcome of this study confirms and corroborates findings from female ferrets primed with a A/Leningrad/134/17/57-based live attenuated cH8/1N1 vaccine followed by vaccination with an AS03-adjuvanted cH5/1N1 split virus vaccine 10.

## 1. Introduction

Influenza viruses are a major cause of disease and mortality in the human population [1]. Current influenza virus vaccines can protect from infection if they are well matched to the circulating virus strains. However, influenza viruses constantly mutate and evade the adaptive immune response by introducing changes in the immuno-dominant head domain of their major surface glycoprotein, hemagglutinin (HA). These changes lead to a loss of protection and can only be partially counteracted by annually updating the vaccine strains because the circulating viruses can change after a new vaccine has already been manufactured [2]. In addition, current seasonal influenza virus vaccines do not protect from potentially pandemic influenza viruses that could emerge from the animal reservoir.

One possible way to address these issues is the development of novel vaccines that elicit antibodies against conserved viral epitopes like the HA stalk domain [3]. Since this part of the HA is immuno-subdominant, immunogens have to be specifically designed to potently induce antibodies against the HA stalk. We have developed a vaccination strategy that uses chimeric HAs (cHA) as antigens [4]. These cHAs consist of exotic avian head domains (to which humans are naïve) and the conserved HA stalk domains of seasonal influenza viruses. The immune system preferentially induces antibodies against epitopes that it had been previously exposed to (consistent with the principle of “original antigenic sin”) [5]. Therefore, repeated vaccination with cHAs and with different HA head domains and the same HA stalk domain can induce high levels of antibodies against the conserved HA stalk domain [6,7,8,9].

Since these cHAs retain the functionalities of wild type HAs (sialic acid binding and fusion activity), live-attenuated vaccine (LAIV) platforms can be employed by this approach [10]. LAIVs have potential additional benefits over parenteral vaccination strategies with inactivated vaccines. These vaccines are applied intranasally and replicated in the upper respiratory tract without causing disease and can, therefore, induce mucosal immunity at the site of entry for influenza viruses. In addition, the replicating vaccine virus can induce T cell responses, which is an advantage usually not associated with inactivated influenza virus vaccines [11].

We have previously tested a cHA LAIV based on the cold-adapted Leningrad vaccine strain, which is followed by an AS03-adjuvanted, inactivated cHA split vaccine immunization strategy in female ferrets with encouraging results [10]. In this study, we used an LAIV with a different cold-adapted backbone known as the Ann Arbor vaccine strain used in the licensed seasonal vaccine FluMist and manufactured and distributed by MedImmune [12]. The inactivated vaccine (IIV) was not a split virion product, but an in-house generated inactivated whole virus formulation. In addition, male ferrets were used instead of female ferrets in this study to test the efficacy of our influenza virus vaccination regimen to determine if the ferret model recapitulates gender differences in immune responses observed in humans. Previous clinical studies have revealed that, in general, women have a more robust immune response than men to the influenza virus vaccine [13,14] with testosterone levels inversely correlated with serum antibody responses in men [15]. We found that the cHA expressing the Ann Arbor-based LAIV was attenuated in vitro and was safe in vivo. The vaccination strategy successfully induced HA stalk-specific antibodies and reduced viral titers after a challenge with a pandemic H1N1 strain. The outcomes were very similar to the study performed with the Leningrad LAIV-AS03-split virus based study in female ferrets. This further supports the development of a universal influenza virus vaccination strategy that relies on an LAIV prime and shows the robustness of the approach.

## 2. Results

### 2.1. cH8/1N1 Live-Attenuated Vaccine Virus has a Temperature Sensitive Phenotype

**Figure 1:**
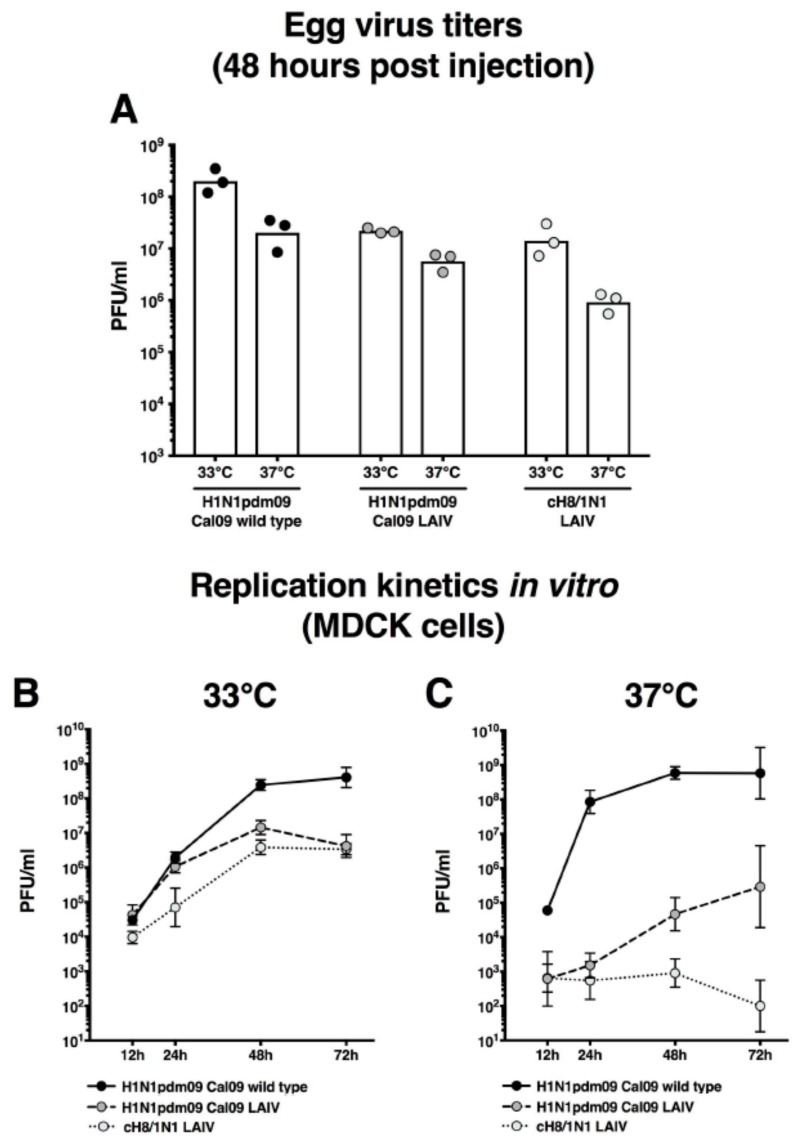
In vitro characterization of the LAIV virus. (**A**) Wild type pandemic H1N1 virus, an LAIV expressing the surface glycoproteins of pandemic H1N1 (dark gray circles), and an LAIV expressing cH8/1N1 (light gray circles) were grown in embryonated chicken eggs at either 33 °C or 37 °C. Virus titers were then determined by using a plaque assay. Each point indicates a biological replicate (individual eggs). Replication kinetics for the three viruses were assessed on MDCK cells at 33 °C (**B**) or 37 °C (**C**) at 12 h, 24 h, 48 h, and 72 h. Each point indicates the geometric mean of three replicates. The error bars show the 95% confidence intervals.

To test the temperature sensitivity of the live-attenuated Ann Arbor virus backbone expressing the cH8/1 and N1 surface glycoproteins (cH8/1N1 LAIV), 100 plaque forming units (PFU) were injected into eggs. The eggs were then incubated at either 33 °C or 37 °C for 48 h. As controls, additional eggs were injected with a recombinant PR8-based H1N1 virus (Cal09) or with a virus consisting of the live-attenuated backbone and the surface glycoproteins of Cal09 (caCal09). The wild-type Cal09 virus showed satisfactory growth at 33 °C with a titer of approximately 2 × 10^8^ PFU, but incubation at 37 °C resulted in a 10-fold reduction of the viral titer (Figure 1A). While human influenza viruses generally grow well at these temperatures, it has been previously described that 2009 pandemic H1N1 isolates grow more efficiently at lower temperatures [16]. The caCal09 virus grew to a lower titer at 33 °C (2.2 × 10^7^ PFU/mL) and was further attenuated at 37 °C (5.7 × 10^6^ PFU/mL). The cH8/1N1 LAIV strain grew to a similar titer as caCal09 at 33 °C (1.4 × 10^7^ PFU/mL) and was even more attenuated when incubated at 37 °C (9.2 × 10^5^ PFU/mL).

To study the growth kinetics in more detail, time course experiments were performed on Madin Darby Canine Kidney (MDCK) cells. At 33 °C, the cH8/1N1 LAIV showed a delayed growth and an approximately 100-fold lower peak titer compared to Cal09 (Figure 1B). The effect was even more pronounced when growth was tested at 37 °C. While Cal09 still reached a peak titer of 5.8 × 10^8^ PFU/mL after 72 h, the titer of cH8/1N1 LAIV reached a low maximum titer at 48 h (9 × 10^2^ PFU/mL) and then decreased to only 100 PFU/mL by 72 h (Figure 1C). These results confirmed the desired temperature-sensitive phenotype of the vaccine strain virus.

### 2.2. cH8/1N1 LAIV Is Attenuated In Vivo

**Figure 2:**
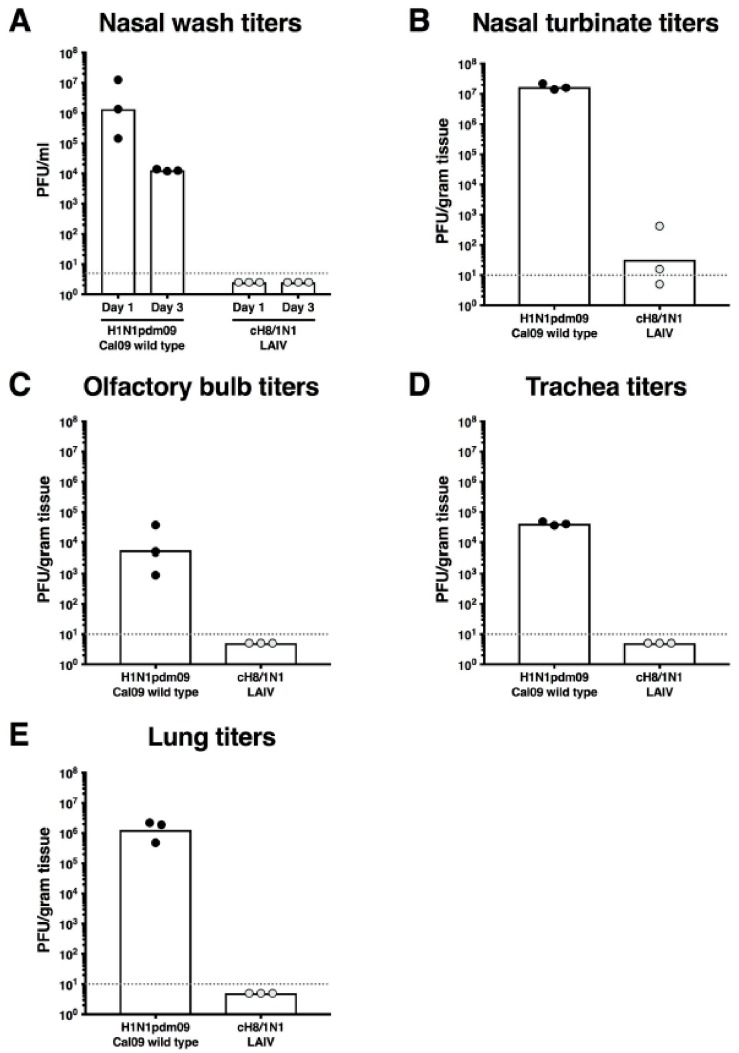
In vivo characterization of the LAIV virus. The level of replication of the cH8/1N1 LAIV (light gray circles) virus was compared to the replication of a wild type pandemic H1N1 virus (black circles) in three ferrets. Viral titers were measured in nasal wash titers (**A**) on days 1 and 3 post-infection. After euthanizing the ferrets on day 4 post-infection, viral titers were measured in the nasal turbinates (**B**), olfactory bulbs (**C**), tracheas (**D**), and lungs (**E**).

Afterward, we tested cH8/1N1 LAIV for pathogenicity in ferrets. Wild-type Cal09 was used as a comparison because it is known to replicate well in ferrets and to cause symptomatic infection. The animals were infected with 1 × 10^6^ PFU intranasally and nasal washes were collected on days 1 and 3 after infection. On day 4 after infection, the ferrets were euthanized and tissues including nasal turbinates, olfactory bulb, trachea, and lung were collected for viral titration. As expected, Cal09 showed good replication in the nasal washes with titers of 1.4 × 10^6^ PFU/mL on day 1. The wild-type virus was also isolated from all tissues of the upper and lower respiratory tract as well as the olfactory bulb (Figure 2). cH8/1N1 LAIV could not be detected in the nasal washes or in any of the tissues except for nasal turbinates (two out of three ferrets). While the titers measured in the nasal turbinates were low, they indicate that low level replication of the virus still occurred until the fourth day in at least two of the ferrets. Based on this outcome, it was concluded that the LAIV was attenuated in vivo and could be considered safe for use in vaccination experiments.

### 2.3. cHA-Based Vaccination with an LAIV Prime Followed by an IIV Boost Regimen or IIV Prime-Boost Regimen Elicit Equivalent Stalk Antibody Responses

**Figure 3:**
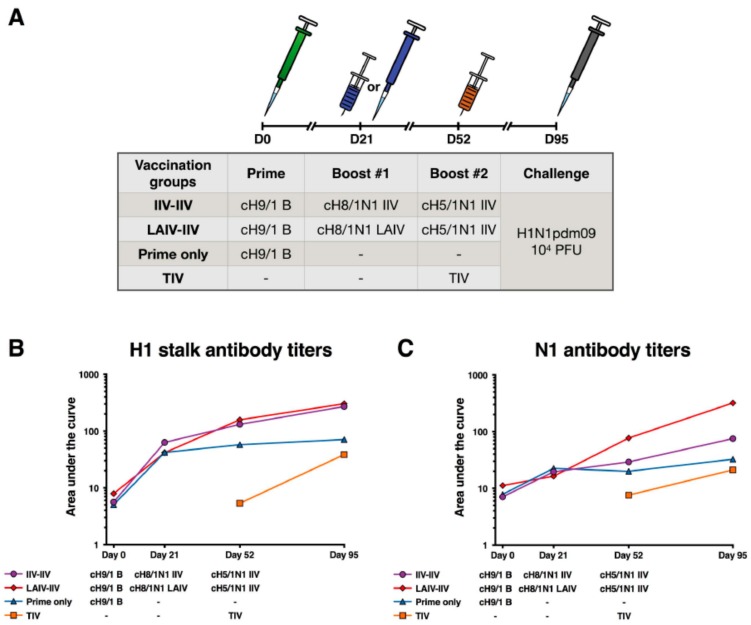
Vaccination strategy and antibody titers measured by ELISA. (**A**) The time line indicates the timing of the vaccinations and the viral challenge. The table shows the corresponding vaccinations for each time point. Serum antibody titer levels were measured by ELISA against the H1 stalk (**B**) and N1 (**C**). The IIV-IIV group is shown as purple circles, the LAIV-IIV group is shown as red diamonds, the prime only group is shown as blue triangles, and the TIV group is shown as orange squares. The y-axis shows the area under the curve and the x-axis shows different time points. Each point shows the geometric mean titer for four animals.

We designed a vaccination experiment in ferrets to test if a cH8/1N1 LAIV prime followed by a cH5/1N1 IIV boost would elicit antibodies against the HA stalk domain and the N1 neuraminidase (Figure 3A). In parallel, a group that received two IIVs (cH8/1N1 followed by cH5/1N1) was tested. As indicated earlier, the LAIV used was based on the Ann Arbor strain and the IIVs used were formalin inactivated whole virus preparations. Both groups were primed with an influenza B virus that expressed a chimeric group A HA (cH9/1) to provide a baseline HA stalk specific humoral immunity without eliciting an influenza A virus specific T cell response that could interfere with a live virus challenge. Ferrets that only received the prime were included as a control group. Lastly, a group of ferrets that received a single shot of the seasonal trivalent influenza virus vaccine (TIV) was included as a standard-of-care control group.

All ferrets showed increases in HA stalk-specific antibodies after the cH9/1 B virus prime (Figure 3B). Both the cHA LAIV and the cHA IIV showed further increases in antibody titers after both cH8/1N1 and the cH5/1N1 vaccination. An increase in HA stalk antibodies was also observed in the TIV group, but the final titers were lower than the titers observed in the cHA vaccination groups. Similar vaccination responses were observed against the N1 neuraminidase (NA). However, the titers against N1 were higher in the group that received LAIV followed by IIV compared to the other groups (Figure 3B). This could have been caused by a higher abundance of NA after active viral replication compared to the fixed amount of NA in inactivated vaccine preparations.

### 2.4. LAIV-IIV Vaccination Was Most Effective in Reducing Viral Loads after the Pandemic H1N1 Virus Challenge

To test the level of protection conferred by the two vaccination strategies, all ferrets were challenged intra-nasally with 1 × 10^4^ PFU of a pandemic H1N1 virus six weeks after the last vaccination. A group of four ferrets was included to measure viral replication after the challenge in the absence of vaccination-induced immunity. Nasal washes were collected on days 1 and 3 after infection. All ferrets were euthanized on day 4 post infection and tissues were collected for viral titer quantification.

**Figure 4:**
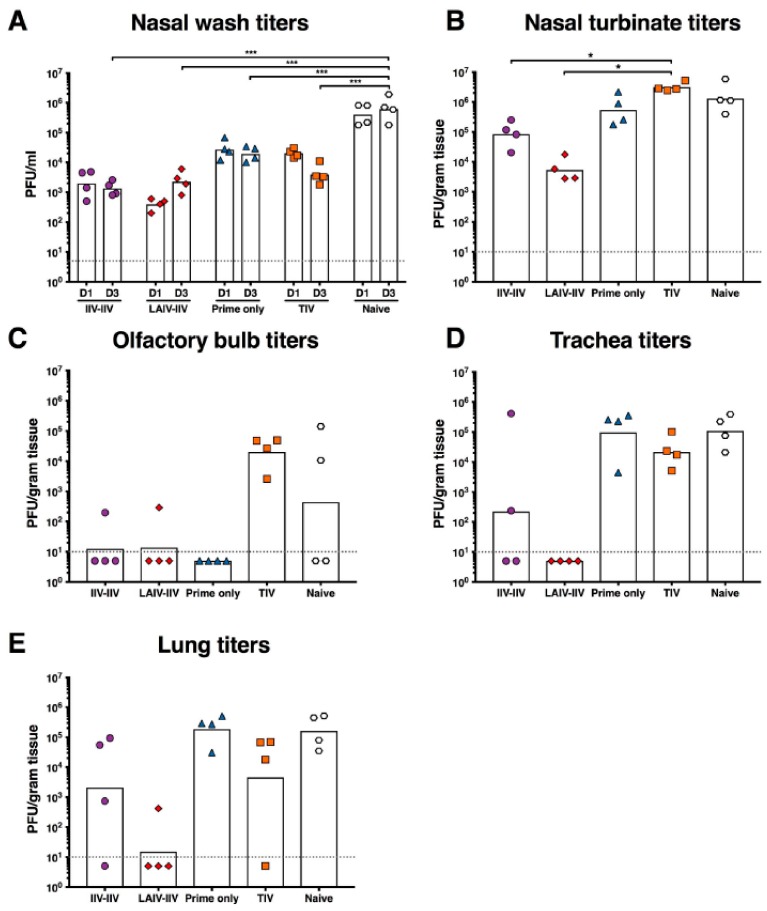
Viral titers after pandemic H1N1 challenge. Viral titers were measured in nasal wash titers (**A**) on days 1 and 3 post-challenge. After euthanizing the ferrets on day 4 post-challenge, viral titers were measured in the nasal turbinates (**B**), olfactory bulbs (**C**), tracheas (**D**), and lungs (**E**). The IIV-IIV group is shown as purple circles, the LAIV-IIV group is shown as red diamonds, the prime only group is shown as blue triangles, the TIV group is shown as orange squares, and the naïve (unvaccinated) control group is shown as white hexagons. Each point indicates an individual animal. The statistical significance between groups is indicated by asterisks (* *p* ≤ 0.05; *** *p* ≤ 0.001).

All vaccinated ferrets showed a reduction in nasal wash titers on days 1 and 3 post infection compared to naïve control ferrets (Figure 4A). The greatest reductions in viral loads were observed in the LAIV-IIV (day 1: 1023.2-fold; day 3: 271.3-fold) and the IIV-IIV (day 1: 204.3-fold; day 3: 460.6-fold) vaccinated groups. 

On day 4, the LAIV-IIV vaccinated ferrets showed a substantial reduction in viral titers in the nasal turbinates (244.8-fold) compared to the naïve control (Figure 4B). There was also an observable reduction in viral titers in the IIV-IIV vaccination group (15.7-fold) while no substantial decrease in viral load was observed for neither the prime only nor the TIV vaccination groups.

Viral replication in the olfactory bulbs in ferrets can be observed with some influenza virus strains including pandemic H1N1 isolates. Two of the naïve control ferrets showed high viral titers in the olfactory bulbs while no virus was detected in the other animals (Figure 4C). All animals in the TIV group showed robust and increased (over naive) viral replication in the olfactory bulb, which is a phenomenon that was previously observed in another study [10]. One ferret in the IIV-IIV and LAIV-IIV groups had low detectable viral titers in the olfactory bulbs.

In the lower respiratory tract, LAIV-IIV vaccinated animals showed the best level of protection against virus replication. No virus was detected in the trachea of animals in this group while high titers were observed in naïve animals (geometric mean titer: 1.1 × 10^5^ PFU/mL) (Figure 4D). Only one ferret in the LAIV-IIV group had low detectable viral titers in the lung (Figure 4E). Some protection was also observed in the lower respiratory tract of IIV-IIV vaccinated ferrets, but a large variability in titers was detected between individual animals. Ferrets that received either TIV or prime only did not show a substantial decrease in virus replication (Figure 4D,E).

## 3. Discussion

Influenza viruses remain a serious threat to global public health. Their ever-changing nature necessitates constant updating of vaccines and annual revaccination. This problem can be addressed by the development of novel vaccines that can broadly protect against a wide variety of influenza virus strains. A universal influenza virus vaccine based on chimeric HAs was previously shown to successfully elicit broadly reactive antibodies and confer broad protection in mice and ferrets [6,7,8,9,10,17]. These hemagglutinin stalk-reactive antibodies induced by our universal influenza virus vaccination regimen neutralized the influenza virus in vivo by Fc-dependent mechanisms [18,19,20].

We have previously published that a vaccination strategy using a chimeric HA expressing LAIV based on the Leningrad vaccine strain as prime in combination with an adjuvanted chimeric HA split virus vaccine can protect female ferrets from a challenge with pandemic H1N1 virus [10,21]. In this study, we aimed to confirm those findings by using a different cHA expressing LAIV based on the Ann Arbor vaccine strain and a whole inactivated virus vaccine in male ferrets [22,23].

The cH8/1N1 LAIV in the Ann Arbor backbone showed temperature-dependent attenuated growth characteristics compared to a wild type virus in both eggs and cell culture. Importantly, the LAIV strain showed substantially reduced viral titers compared to a wild type infection in ferrets and no overt adverse vaccination-related events were observed. These findings further confirm that LAIV vaccines that express cHAs can be successfully attenuated with conventional vaccine strain backbones and are safe in animals.

Vaccination with the LAIV and IIV vaccines induced HA stalk-specific and N1 neuraminidase specific antibodies in vaccinated ferrets. Similar to our previous findings, we show in this paper that LAIV-IIV vaccination significantly reduced viral replication in the upper respiratory tract (Figure 4A,B) and reduced replication of the challenge virus in the lower respiratory tract. Although the LAIV-IIV and even the IIV-IIV vaccination regimen did not confer sterilizing immunity, the degree by which these vaccination regimens reduced viral replication would significantly reduce the shedding of the influenza virus from the upper respiratory tract, which would translate to reduce transmission of the influenza virus. The reduced levels of viral replication in the lower respiratory tract would also translate to reduced clinical disease following natural infection. In conclusion, data obtained from this study that used different vaccines and male instead of female ferrets shows that the observed effects are robust and reproducible and further supports the validity of this vaccination rationale to develop a truly universal influenza virus vaccine that protects from all influenza A viruses and influenza B viruses [24].

## 4. Materials and Methods

### 4.1. Viruses and Vaccines

The challenge virus (A/California/04/09, Cal09) was grown in 8-day to 10-day old specific pathogen free, embryonated chicken eggs at 37 °C for 2 days. The LAIV strain was generated by standard reverse genetics using plasmids that expressed the 6 internal proteins of the Ann Arbor vaccine strain (A/Ann Arbor/6/60), which is a plasmid expressing cH8/1 (H8 head domain from A/mallard/Sweden/24/02, H1 stalk domain from A/California/04/09) and a plasmid expressing N1 (from A/California/04/09) [4,10,23,25]. The LAIV stock virus was grown at 33 °C for 3 days. The IIV strains were also generated by reverse genetics, but contained the internal segments of PR8 (A/Puerto Rico/8/34). The HA plasmid for the cH8/1 virus strain was identical to the one used for the LAIV and the plasmid for the cH5/1 virus expressed the H5 head domain from A/Vietnam/1203/04 with the H1 stalk domain from A/California/04/09. Both viruses were generated with the wild type N1 (A/California/04/09) plasmid. The PR8 re-assortant strains were grown at 37 °C for 2 days. The allantoic fluid was harvested, purified by ultra-centrifugation through a sucrose cushion, and incubated with 0.03% of formaldehyde at 4 °C for 72 h to inactivate the viruses. The HA content of the virus vaccine preparations was measured as previously described and each dose was adjusted to contain 5 µg of HA [26]. The B-cH9/1 virus used for priming of the ferrets was based on a recombinant B/Yamagata/16/88 backbone with an HA that features the HA stalk domain from an H1N1 strain PR8 and the head domain from an H9N2 strain A/guinea fowl/Hong Kong/WF10/99 [8,27]. The chimeric influenza B virus stock was grown at 33 °C for 3 days.

### 4.2. Animals

Four month old, castrated, and descended male Fitch ferrets were confirmed seronegative for circulating H1N1, H3N2, and B influenza viruses prior to the purchase from Triple F Farms (Sayre, PA, USA). Ferrets were housed in Plas-Labs isolation units with free access to food and water. All animal experiments were conducted using protocols approved by the Icahn School of Medicine at Mount Sinai (ISMMS) Institutional Animal Care and Use Committee (IACUC; Protocol #LA12-00170). Animals were anesthetized by intramuscular administration of ketamine/xylazine for all experimental manipulations.

### 4.3. LAIV Phenotypic Analysis and In Vivo Pathogenicity Testing

To examine the temperature sensitivity of the LAIV, specific pathogen-free embryonated chicken eggs were inoculated with 100 PFU of the indicated virus. At 48 h post-inoculation, the eggs were chilled and then allanotic fluid was harvested to examine viral replication by a hemagglutination assay and to quantify virus titers by plaque assays on MDCK cells. To examine the replication phenotypes of the LAIV, MDCK cells were infected at a multiplicity of infection (MOI) of 0.01. Following infection, the MDCK cells were cultured in serum-free MEM supplemented with 1 µg/mL *N*-tosyl-l-phenylalanine chloromethyl ketone (TPCK)-treated trypsin. At the indicated time points, supernatants from the cell cultures were collected for quantification of virus titers by plaque assays on MDCK cells. Nasal washes were then taken on day 1 and 3 post-infection and weight was monitored daily (data not shown). On day 4 post-infection, animals were sacrificed and tissue samples from the lung (upper right lobe), olfactory bulb, and nasal turbinates were collected for subsequent quantification of virus titers by plaque assays on MDCK cells. 

### 4.4. Ferret Vaccination and Challenge

Four ferrets were used in each vaccination group. All animals except for the TIV group were primed with 1 × 10^6^ PFU of cH9/1 B virus. On day 21 post-prime, one group (IIV-IIV) received 5 μg (normalized to HA content) of the inactivated cH8/1N1 vaccine and one group (LAIV-IIV) received 107 PFU of the cH8/1N1 LAIV. On day 52 post-prime, both the LAIV-IIV and the IIV-IIV groups received 5 μg of the inactivated cH5/1N1 vaccine and the TIV group received a standard 15 μg per strain seasonal influenza virus vaccine. On day 95 post-prime, the animals were intra-nasally challenged with 1 × 10^4^ PFU of the pandemic H1N1 (A/California/04/09) virus. Ferrets were bled on days 0, 21, 52, and 95 for serology. Nasal washes were collected on days 1 and 3 post-challenge. The animals were euthanized on day 4 post challenge and tissues were collected for viral titer quantification.

### 4.5. ELISAs

High binding 96-well plates (Immulon 4HBX) were coated with 200 ng of either cH6/1 (H6 head domain from A/mallard/Sweden/81/02, H1 stalk domain from A/Puerto Rico/8/34 with a Strep-TagII purification tag) or N1 (from A/California/04/09 with a hexahistidine purification tag) in 50 μL of sodium bicarbonate buffer (pH 9.4) and incubated at 4 °C overnight [28,29]. Plates were blocked with PBS-T (PBS [phosphate buffered saline] containing 0.1% Tween 20) containing 3% goat serum (Gibco) and 0.5% milk powder. The blocking solution was removed after 1 h incubation at room temperature and samples serially diluted in blocking solution were added. Plates were washed 3× with PBS-T after 2 h of incubation and horse radish peroxidase (HRP) labeled as a secondary antibody (Alpha Diagnostic International, #70530, San Antonio, TX, USA) was added to each well at a 1:3000 dilution in 50 μL of blocking solution. After 1 h of incubation, plates were washed 4× with PBS-T and developed with 100 μL of SigmaFast OPD (o-Phenylenediamine dihydrochloride; Sigma, St. Louis, MO, USA) and stopped after 10 min with 3 M HCl. Plates were read at an optical density of 490 in a plate reader (Synergy H1, BioTek Instruments, Winooski, VT, USA). Data was then analyzed in Microsoft Excel (Office 2010, Redmond, WA, USA) and GraphPad Prism (version 7.0d, Mac version, LaJolla, CA, USA).

### 4.6. Plaque Assays

MDCK cells were cultured in complete Dulbecco’s Modified Eagle's Medium (cDMEM) in a 37 °C incubator with 5% CO_2_. Six-well plates were seeded with approximately 6.5 × 10^5^ cells and incubated overnight. After confirming that each well was covered with a confluent monolayer of cells, cDMEM was removed and cells were washed with PBS. After washing, each well of a plate was incubated with 10-fold serially diluted virus or virus containing specimens for 1 h at either 33 °C (for LAIV virus strains) or 37 °C (for wild type virus strains). Plates were then washed with PBS and an overlay containing minimal essential media (MEM), 2% Oxoid agar, TPCK-treated trypsin, and DEAE (Diethylaminoethyl) dextran was added. Plates were incubated for 48 h to 72 h until plaques had developed. Plaques were visualized by immunostaining or crystal violet staining.

### 4.7. Ethics Statement

All animal procedures were performed in accordance with the Icahn School of Medicine at the Mount Sinai Institutional Animal Care and Use Committee (IACUC).

### 4.8. Statistical Analysis

Data was analyzed and graphed in GraphPad Prism 7. Viral titers in nasal washes were compared in a 2-way ANOVA with a Tukey post-test for multiple comparisons. Viral titers in tissues were compared in an ordinary one-way ANOVA with a Tukey post-test for multiple comparisons. Statistical significance is indicated directly within the figures by the addition of asterisks.

## 5. Conclusions

In this study, we examined the efficacy of our sequential immunization approach vaccines containing chimeric hemagglutinin (cHA) to confer protection against influenza virus infection. Male ferrets prime immunized with a cH8/1N1-expressing live-attenuated influenza virus (LAIV) vaccine based on the A/Ann Arbor/6/60 strain, followed by a booster immunization with a non-adjuvanted, formalin-inactivated cH5/1N1 whole virus vaccine. This vaccination group was compared to a second group that received a cH8/1N1 inactivated vaccine followed by a cH5/1N1 inactivated vaccine. Whereas both immunization regimens conferred protection to the upper respiratory tract against replication of A(H1N1)pdm09 virus, ferrets that were immunized with the LAIV were better protected against lower respiratory tract viral replication. These results support the further development of chimeric hemagglutinin-based vaccination strategies, and their translation to clinical trials.
